# Changes to utilization and provision of health care in German GP practices during the COVID 19-pandemic: Protocol for a mixed methods study on the viewpoint of GPs, medical practice assistants, and patients

**DOI:** 10.1371/journal.pone.0279413

**Published:** 2023-04-13

**Authors:** Susanne Doepfmer, Kemal Akdenizli, Hiwa Dashti, Christoph Heintze, Frank Kaden, Lisa Kuempel, Doreen Kuschick, Natascha Medrow, Andrea Neidhardt-Akdenizli, Susanna Otto-Gogoll, Isabelle Reicherdt, Lydia Steenweg, Kahina Toutaoui

**Affiliations:** 1 Institute of General Practice, Charité—Universitätsmedizin Berlin, Corporate Member of Freie Universität Berlin and Humboldt-Universität zu Berlin, Germany; 2 Member of the RESPoNsE Practice Advisory Board, Practice in Berlin, Germany; 3 Member of the RESPoNsE Practice Advisory Board, Practice in Brandenburg, Germany; Duke University, UNITED STATES

## Abstract

**Introduction:**

During the COVID-19 pandemic, general practitioners (GPs) continued to be a main point of contact for patients. For GP practices, it was and still is a challenge to meet constantly changing requirements due to the various phases of the pandemic. The aim of the study is to explore retrospectively the subjective experience with supply and utilization of health care services from the perspective of general practitioners, medical practice assistants and patients, in particular regarding instances of underutilization of services for non-Covid related conditions, adjustments due to the pandemic, and the appropriateness of care.

**Methods:**

The study is carried out within the RESPoNsE research practice network in three of Germany’s federal states: Berlin, Brandenburg, and Thuringia (RESPoNsE—Research practice network east). The study follows a convergent mixed method design, and consists of the following sections: a) two anonymous paper-based questionnaires filled out by GPs and medical practice assistants (MPAs), at an interval of 12 to 18 months; b) in-depth qualitative interviews conducted among a subgroup of GPs and MPAs; c) anonymous paper-based questionnaires among patients of participating practices. The idea for the study was derived from discussions with the practice advisory board of the RESPoNsE network. The themes and issues to be explored in the surveys and interviews are developed and discussed in the practice advisory board, the patient advisory board, and with interested MPAs. The questionnaires will be analyzed descriptively, exploring the effect of demographic variables. Qualitative content analysis is used to analyze the data from the interviews and focus groups.

**Discussion:**

The study focuses on the conditions of GP care during the COVID-19 pandemic. A broad insight is provided as GPs and MPAs, as well as patients, are involved. It provides the opportunity to express needs and concerns. The results can support future discussions on lessons learned from the pandemic and necessary changes in health care delivery.

**Trial registration:**

Trial registration at the German Clinical Trials Register: DRKS00028095.

## Introduction

Primary care plays a crucial role during the COVID-19 pandemic [1, 2].

While hospital care and the concern around potential insufficient intensive care resources were very much a focus in the earlier stages of the pandemic, it was the GP practices who usually (and, again, were) the first point of contact for the patients. Practices often had to decide who needed hospital care; they also provided and monitored patients with COVID-19 at home while providing continuous care for all other patients [3–5].

In Germany, 80 to 85 percent of the patients suffering from Covid-19 received medical care in the ambulatory care sector [6]. Important characteristics of the German health care system as a framework for GP practice organization are described in Box 1.

**Box 1. Characteristics of the German health care system as a framework for GP practice organization**

Generally

- lack of a gatekeeping system;
- important role of self-governing bodies [7, 8].

General practitioners

- are on average responsible for 2,000 inhabitants;
- see on average 242 patients per week, with an average consultation time of 7.6 minutes;
- are usually self-employed and work in large part in a single-handed practice;
- usually rely on a personal communication infrastructure with colleagues, other specialists, and nursing facilities;
- largely decide autonomously about staff, working hours, and the organization of their practices [7, 9].

Medical practice assistants

- (also called health care assistants, or “Medizinische Fachangestellte” in German)
- work under the legal responsibility of the GPs;
- go through a three-year vocational training program;
- are qualified for office and management duties as well as basic clinical tasks, such as measuring blood pressure and taking blood samples;
- may take over additional tasks including injections, vaccinations, or home visits, according to the “Agreement on the delegation of medical services to non-medical staff in ambulatory healthcare” [10–12].

The pandemic resulted in several spontaneous and unsystematic changes in the supply and uptake of health care services, affecting general practitioners (GPs), medical practice assistants (MPAs), and their patients [13, 14].

The idea for the study protocol presented here was developed in a practice advisory board meeting of the practice-based research network RESPoNsE (for details on RESPoNsE, see Box 2).

**Box 2. Context of the study: Practice-based research network “RESPoNsE”**

- “RESPoNsE” is one of six regional practice-based research networks (PBRNs) in Germany funded by the Federal Ministry of Education and Research, comprising practices in three of the 16 federal states in Germany: Berlin, Brandenburg, and Thuringia.
- These three states together represent 10% of the German population, with the metropolitan area of Berlin and the small- to medium-sized towns and rural areas of Brandenburg and Thuringia, and a medium to below average range of socioeconomic status with regional differences within the federal states [15–17].
- PBRNs are implemented in many countries and regions to involve primary care practices and patients in identifying relevant research questions [18].
- RESPoNsE aims to recruit and qualify GPs and MPAs for research activities [19].

GPs and MPAs expressed their view that, although they provided a major portion of COVID-19 patients´care, their worries and issues had rarely been featured in medical journals or the press and were not well-known to the general public. Previous research could show that GPs, from early on in the pandemic, rapidly re-organized their practices, from a fast uptake of virtually-delivered care by use of video or telephone to doctor-led triage [1, 20–25], even though they had to cope with scarce, delayed, or confusing information from public bodies, and were not supplied with sufficient personal protective equipment (PPE) [4, 6, 26, 27]. In 2020, Huston et al. found that preparedness and ability to adapt to the new situation varied between GPs of different countries [28], all of them with a very different health care systems to Germany. Evidence of adaptions in German GP practices is scarce. It is mainly from early phases of the COVID-19 pandemic and is based either on small surveys or on qualitative interviews [29–31].

Reports of a deteriorating work climate in German GP practices also date from early phases of the pandemic [32, 33].

Even though the MPAs in Germany also play a crucial role in meeting the new challenges in German GP practices, there have been only few reports on their perspective from early phases of the COVID-19 pandemic, in which concerns are described regarding the uncertain course of the pandemic, worries and a high level of responsibility for the wellbeing of their patients, fear of contracting COVID-19, and a general feeling of not being properly prepared for the challenges of the pandemic, including the necessary organizational adaptations [34, 35]. While, at the beginning of the pandemic, the focus of public interest and reporting was on the situations of intensive care units and the working conditions for hospital nurses [36], only recently have MPAs received more public attention toward the burden they bore [37].

From the beginning of the pandemic, utilization of health care for non-urgent needs declined [38]. Due to a fear of contracting COVID-19, patients avoided medical care wherever possible [39], sometimes with severe consequences, including death from strokes, heart attacks, cancer, and other acute illnesses [40–42]. A longitudinal online-based patient survey could show that, from the perspective of the patients, the supply of medication has broadly not been impaired, but also revealed that it was considerably more difficult to get an appointment with the GP, with results differing according to which phase the pandemic was concurrently in [43]. In the first six months of the pandemic, in particular, patients postponed regular health checks and routine vaccination appointments [44].

At the same time, mental exhaustion, anxiety, and feelings of loneliness and isolation increased among the general population, adding to the challenges faced by GPs and MPAs during the pandemic [45].

For all three groups, the COVID-19 pandemic may change the general view on the appropriateness of care. Before, there was only limited awareness of unnecessary care [46]. Now (during the pandemic) health care providers, and patients had to decide intuitively and without clear guidance or evidence which medical services were truly necessary [14, 47].

Therefore, the pandemic may also provide the opportunity to revisit health care practices that may not be effective or efficient [13, 48].

It is the subjective experience during the pandemic from the three groups involved (GPs, MPAs, and patients) that most likely will shape the future health care landscape.

Our study will add to the existing evidence by providing:

An up to date view of German GPs, MPAs, and patients on health provision and utilization, appropriateness of care, and the perceived burdens more than two years from the beginning of the COVID-10 pandemic;

An integration and comparison of the views of these three groups;

A participatory approach, involving the three groups already in the development of the study, which, therefore, addresses aspects that were identified as relevant by them;

To our knowledge, no study has been reported that describes the COVID-19 pandemic experiences of all three of these groups (GPs, MPAs, and patients) in Germany, and that addresses organizational adaptation needs, supply, utilization, and quality of care within the GP practices, as well as support needs, and concerns and worries.

### Research framework

Integrating the literature search and discussions with GPs and MPAs, we built our study on the “Covid-19 system shock framework” by Hodgins et al., which identified changes to health services, health workface, information systems, medical products and technologies, funding and finance, health system values and health policy and governance as the main dimensions of relevance to health systems’ responses to the pandemic [49], (Table 1).

**Table 1:** The COVID-19 system shock framework and themes in the study.

Concepts from the Covid-19 system shock framework	Themes addressed in our study
Changes to health services	Practice organization
	Change of frequency of services
Medical products and technologies	Telephone and Video consultations Protective equipment
Information Systems	
	Supply, usefulness and processing of information
Governance	
	Extra funding for additional work
Funding and Finance	
	Qualification
Health workforce	Stress
	Job satisfaction
	Team spirit
	Responsibility, motivation, and concern
Values	Appreciation
	Appropriateness of care

## Methods

### Aim

This study addresses the following research question:

What are the views of GPs, MPAs, and patients on the provision and utilization of health services during the COVID-19 pandemic? With particular regard to:

- aspects of underutilization/undersupply of regular care for non-Covid related conditions;
- requirements, practicability and sustainability of adjustments to practice organization and management (for example virtually-delivered care); and
- the appropriateness of regular care services that may have seemed dispensable under pandemic conditions

Furthermore, the three groups involved (GPs, MPAs, and patients), will have the opportunity to express other aspects or concerns experienced during the COVID-19 pandemic.

### Participatory approach

Using a participatory approach, GPs and MPAs, and patients will play an important role in the PBRN in choosing and prioritizing research topics and in assessing the relevance and feasibility of a research project.

For this purpose, a practice advisory board comprising GPs and MPAs, and (separately) a patient advisory board was founded at the two project centres in Berlin and Jena, Thuringia, to reduce the need for long-distance travel. Research ideas and activities are discussed regularly in the board meetings, enabling participation of the three groups (GPs, MPAs, and patients) at every stage of a project. In our view, it is essential to encourage the participation of all groups who are affected by the research activities and by the potential outcomes.

Serving on a study board or advisory council and attending regular meetings with researchers is one of the most active form of engagement is [50]. In addition to the regular commitments to the practice board by a dedicated group of GPs and MPAs willing to attend regular meetings, all other PBRN GPs and MPAs are invited to visit network meetings, which are held quarterly and provide the possibility to discuss research activities and study ideas.

The Covid-19 pandemic is the focus of this study but also an important factor for the readiness to participate in this particular study and generally in the PBRN:

The pandemic may highlight the importance of research in the primary care setting, as could be shown in an increased interest in research [23]. Therefore, the study will also be combined with a new recruitment effort for the PBRN.

At the same time, research activities may add an extra burden to a practice team that already has reached its limit [51].

Board and network meetings have so far been held via video conference due to pandemic-related restrictions. While this also has the advantage of convenience and saving time—and allows the participation of less mobile patients—it may at the same time exclude patients who are not digitally literate. Being fully aware of these issues, our approach is necessarily pragmatic.

The mode of participation in RESPoNsE is to be seen as consultative to collaborative [52]. While the power for decision-making remains with the research team, due to the accountability owed to the funder, nevertheless we believe that there is “the potential for sharing experience, knowledge and the harnessing of multiple perspectives” [53]. How and when best to involve the three groups will be a learning process that will form part of the structural development of the network, and some fluctuation of level and scope of participation is expected, depending on the context and on individual interests [54, 55].

The idea for this study was derived from discussions in the practice advisory board in May 2020. In order to strengthen the participatory approach, the study has been conceptualized in line with the impressions GPs and MPAs have that some aspects of their work during the COVID-19 pandemic had not yet been addressed adequately: concern regarding the ability to maintain the quality of routine care, a lack of resources (both material and personnel), a constant need for operational responsiveness to changing conditions, a lack of support, and confusing or insufficient information from regulatory and professional bodies. These issues were further discussed in a meeting with a group of interested MPAs, invited via the MPA branch of the Association of Medical Professions (“Bundesverband medizinischer Fachberufe”.) Further aspects of this discussion were new challenges in the area of patient contact (aggressive behavior, for example) and the feeling of a lack of respect from patients and the public, consequentially impacting their job satisfaction.

### Study design and setting

The study will be carried out in three German federal states that are covered by the RESPoNsE network (Berlin, Brandenburg, and Thuringia), comprising approximately eight million inhabitants.

Due to the explorative nature of the study, no sample size calculation was undertaken. Every GP in Berlin and Brandenburg who is statutory health insurance registered (approximately n = 4.100) may take part in the study. The response rate will be a secondary outcome, comparing the rates for GPs who already are part of the RESPoNsE network with those who are not. As other surveys in the Thuringia GP practices are taking place at the same time, only those practices that already are members of RESPoNsE (n = 70) are invited to take part in the study. There are no exclusion criteria.

The GPs have received a postal invitation to take part in the study. MPAs may take part in the study independently of the GPs in the same practice but, as we only have information on the GPs working per practice (and not the MPAs), MPAs will only receive information about the study from the GP who received the letter at their practice.

In line with previous work and similar surveys with GPs, we expect a response rate of about 25% [56, 57]. We expect the response rate to be higher among GPs who are already a part of the RESPoNsE network. As we don`t know how many MPAs are working in the practices we will not be able to calculate a response rate for the MPAs.

The letter to the GPs consists of:

- a study information;
- the questionnaire for GPs (to be filled out anonymously);
- the questionnaire for MPAs (to be handed out to interested MPAs in the same practice and filled out anonymously by as many MPAs as are willing to take part in the study—the questionnaire be copied if necessary);
- a prepaid return envelope;
- a response form (S1 Form), asking for their readiness to take part in the survey or in the further portions of the study (to be filled out with contact details and to be sent back separately via fax or mail);
- The GPs and MPAs in Berlin and Brandenburg who are not yet members of the RESPoNsE network are also provided with information on network activities and aims and will be asked to express their interest to be part of the network.

**Fig 1:**
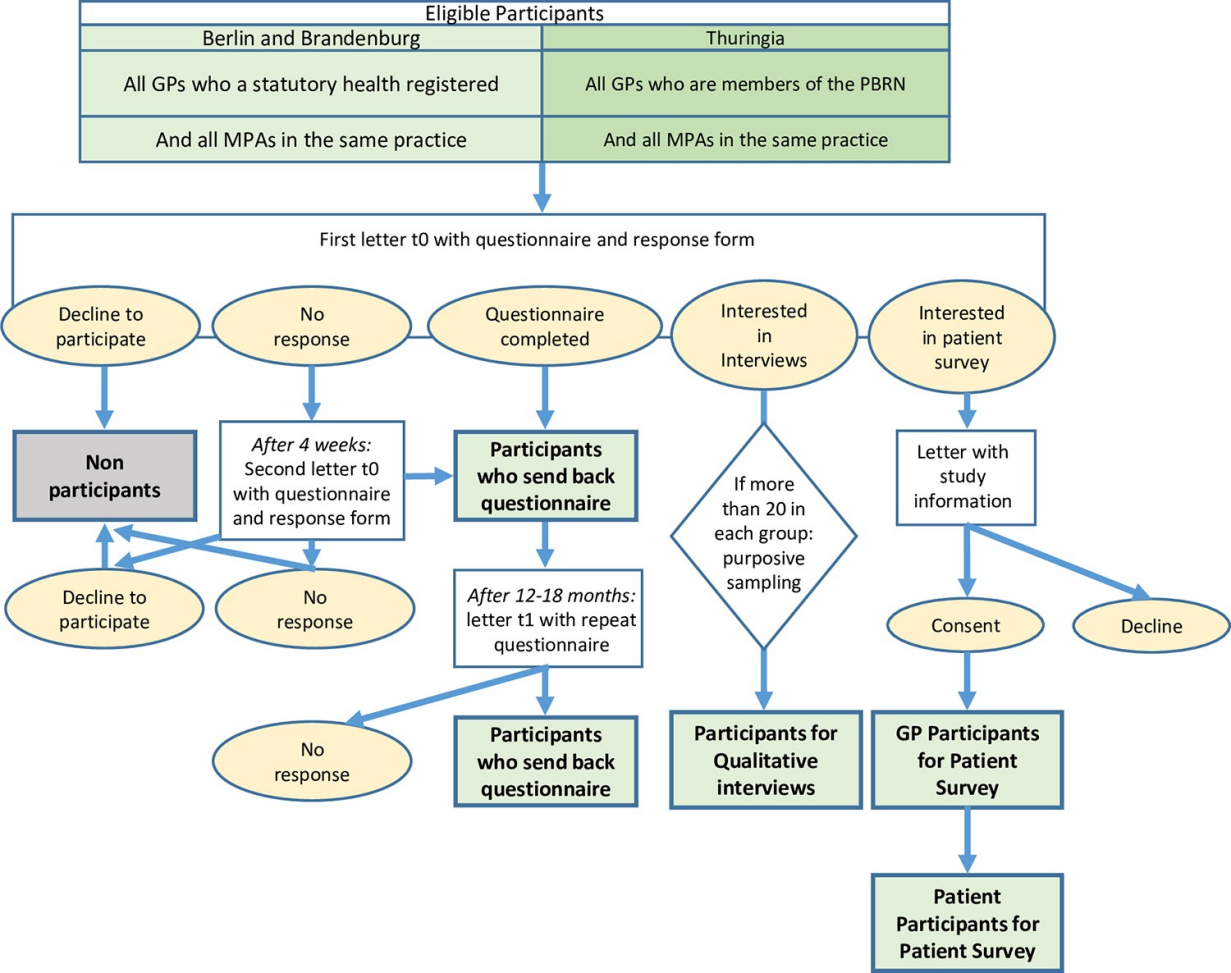
Flow chart of participants.

After 4 weeks the same letter was sent as a reminder to those GPs who had not yet returned their response form (Fig 1).

### Study structure

This convergent mixed methods study includes the following methodological approaches (see Table 2 for a summary):

**Table 2:** Study structure: Purpose and data source.

	Purpose	Data Source
Phase 1 Participatory approach	Collect themes for surveys. Develop surveys.	Practice advisory board. Patient advisory board.
Phase 2 Surveys with GPs, MPAs and patients	Explore experiences of the three groups during the COVID-19 pandemic. Recruit MPAs and GPs for interviews. Recruit GPs and MPAs for the PBRN.	GP and MPAs in three federal states. Patients of participating GP practices.
Phase 3 Qualitative interviews	In-depth exploration of survey results.	GPs and MPAs in three federal states.
Phase 4 Follow-Up Surveys	Repeat survey (GPs and MPAs) to explore views and attitudes one year later.	GPs and MPAs who took part in the first survey.

### Quantitatively: Surveys

The questionnaire for GPs and MPAs was constructed and piloted with the participation of the practice advisory board and addresses aspects of routine health care provision during the COVID-19 pandemic, as well as organizational, technical, and staffing measures, external information, and fears and worries about their work environment during the pandemic.

Most of the questions are the same or equivalent for both GPs and MPAs, including information on demographic and professional characteristics (gender, age, location of practice, professional experience). One thematic block varies between the two groups, allowing for a different focus during the preparatory discussions in the meetings with GPs and MPAs. Except for the demographic questions, all the other items are designed on a five-point Likert scale, supplemented by a “don´t know” category where appropriate (Table 3; S2 and S3 Files).

**Table 3:** Questionnaire for GPs and MPAs (themes, examples, scale).

	Thematic block	Scale
Identical / equivalent to GPs and MPAs	demographic and professional characteristics (gender, age, location of practice, professional experience)	Single choice items
	Changes in frequency for procedures during the coronavirus pandemic (for example scheduled / unscheduled visits, virtually delivered care by use of video or telephone, home visits, routine checks, vaccinations other than Covid-19)	Much less frequent–less frequent–no change–more frequent–much more frequent–don´t know
	*Example statement*: *“How has the frequency for the following service for non-Covid-19 related conditions changed during the pandemic … home visits”*	
	Subjective assessment of the quality of care for chronically ill patients during the corona virus pandemic (for example, changes in quality of care, changes in health conditions of the patients, changes in number of practice visits).	strongly agree–agree—neutral- disagree -strongly disagree -don´t know
	*Example statement*: *“We managed to provide the same quality of care as before”*	
	Personal view on working conditions and job satisfaction during the pandemic (for example, expectations, conflicts within the team, joy at work).	strongly agree–agree—neutral- disagree -strongly disagree
	*Example statement*: *“I am thinking about changing my job”*	
	Requirements/resources in order to maintain health care provision during the pandemic (for example, material, qualification, information).	strongly agree–agree—neutral- disagree -strongly disagree
	*Example statement*: *“In order to maintain the provision of care under pandemic condition we need … better qualified personnel”*	
GPs only	Subjective assessment of usefulness of information by different professional associations and organizations during the pandemic (for example, the General Medical Council, Standing Committee on Vaccination, Federal Centre for Health Education, German College of General Practitioners and Family Physicians).	Very helpful–rather helpful–neutral–rather not helpful–not helpful at all –don´t know
	*Example statement*: *“How would you assess the information given during the pandemic by the… German College of General Practitioners and Family Physicians“*	
MPAs only	Personal anxieties and concerns during the pandemic (for example, stress, risk of infection and health issues, social conflicts, economical burdens).	strongly agree–agree -neutral- disagree -strongly disagree
	*Example statement*: *“What are the primary issues of fears and worries for you during the pandemic… constant stress “*	

Participating GPs and MPAs will be asked to fill in a second questionnaire after 12 to 18 months. In this way, we will explore which views and possible changes in health services persist over time. As the questionnaires will be anonymous, the comparison will be made on an aggregated level.

The GPs who participated in the first survey will also be asked to cooperate in the patient survey. Those who indicate an interest on the response form will be contacted again with further information on the proceedings and will be asked to consent into participating in the study. From a previous study (not yet published), we expect about 50 to 100 GPs to take part in this portion of the study. Participating GPs may indicate whether they expect to successfully distribute questionnaires for 50 or 100 patients during the standardized study period of four weeks. Questionnaires will be sent to the practices accordingly to be distributed among patients who sit in the waiting room. Patients will be provided with information on the study, asked to fill in the questionnaire anonymously, and to place it in a pre-prepared box. After the given study period, the practice team will send back the questionnaires using a prepaid envelope.

Inclusion criteria are:

- At least 18 years old or more
- Able to read and fill out a German questionnaire

### Qualitatively: Focus groups and interviews

Two focus groups with members of the patient advisory board were performed to develop the patient questionnaire. The format for the groups was based on a semi-structured focus group discussion guide, building on preliminary discussions with the patient and practice advisory board and a literature search, as knowledge bases.

The focus groups were conducted using an online format, and were recorded, transcribed verbatim, and analyzed thematically using a combined deductive-inductive approach.

Emerging themes were:

- Changes to utilization and perceived changes of provision of health services in a GP practice.
- Perceived roles of GPs and practice teams during the pandemic.
- Information needs during the pandemic and perceived quality of information given by GPs
- Concerns and fears during the pandemic.

Qualitative interviews with GPs and MPAs will be performed after the GP and MPA-questionnaires are analyzed and will provide a more in-depth exploration of some aspects of the questionnaire.

GPs and MPAs who indicated their interest are eligible to take part in the interviews. There are no exclusion criteria. We aim to conduct 15 to 20 interviews in each group or until thematic saturation is reached independently of both groups, using a new information threshold of <5% [58]. Purposive sampling will be applied if more than 20 GPs and 20 MPAs are interested in taking part in this study [59]. All interviews will be held by the same member of the research team and will last 30 to 40 minutes.

The semi-structured interview guide is developed according to the framework by Kallio et al and will build on the preliminary discussions with GPs and MPAs, results of the questionnaire and a literature search as knowledge bases, building a preliminary interview guide addressing main themes and using follow-up questions, performing internal testing and field testing in a pilot phase and finalizing a complete semi-structured interview guide [60].

It may include the following aspects:

- Lessons learned / best practice models
- Identified support needs
- Challenging situations
- Sustainability of changes in provision and organization of care during the pandemic.

Interviews will be audio-recorded and transcribed verbatim. Any identifying details will be removed. Written informed consent will be obtained in advance.

### Mixed methods: Integration and triangulation

In this study, different perspectives, methods, and time points have to be combined and integrated in a meaningful way. Integrating Quantitative and Qualitative Methods has a long tradition in primary care studies [61, 62].

We draw on the definition of Johnson et al. for mixed methods research: “Mixed methods research is the type of research in which a researcher or team of researchers combines elements of qualitative and quantitative research approaches (e.g., use of qualitative and quantitative viewpoints, data collection, analysis, inference techniques) for the broad purposes of breadth and depth of understanding and corroboration” [63].

**Fig 2:**
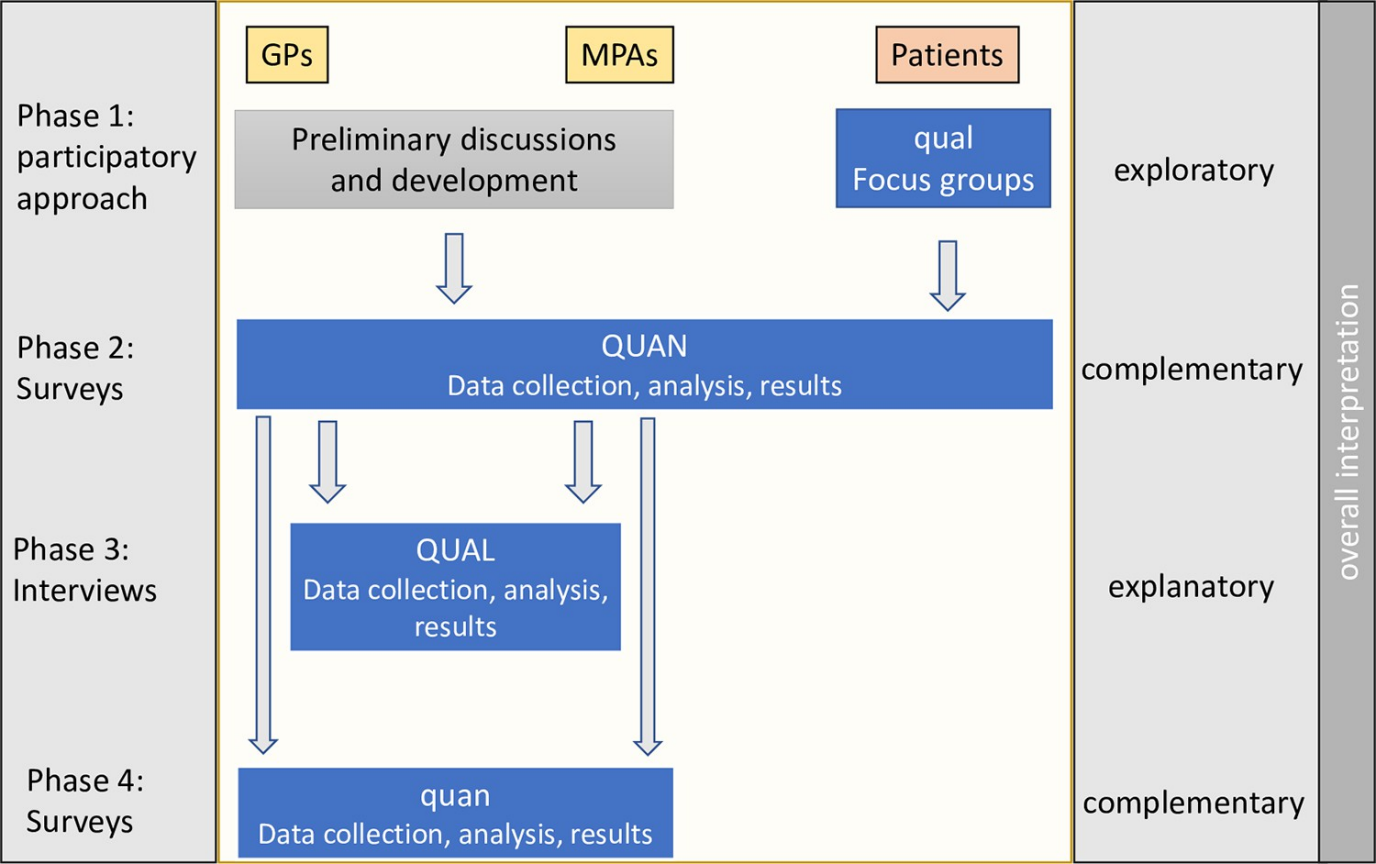
Mixed methods design.

The purposes of our mixed method approach are development and complementarity, using a convergent design and building on the notion from Creswell and Plano Clark that a mixed methods design should be labelled according to the intent of the design [64–66] (Fig 2).

Integration will use triangulation methods [62] and will address:

- The perspective of the members of the research team (Investigator-Triangulation):

The research team consists of two senior researchers who are GP and have a long-standing experience in qualitative and quantitative research, a junior researcher who is in training as a GP and has a qualification in qualitative methods, health care scientists with experience in qualitative and quantitative research (one of them with a background in nursing) and of the members of the practice advisory board who are GPs or MPAs in Berlin and Brandenburg. Quantitative analysis will be supported by biostatisticians. Every step of the study will be discussed within the research team to integrate the different perspectives and backgrounds, with the aim of reducing observer bias.

- The perspective of the three groups involved and different time points for two of the groups (Data-Triangulation):

The three groups (GPs, MPAs, and patients) were involved in the development of the research question and/or the identification of themes to be addressed in the study. The results of the study portions will be compared between the groups, where applicable. For GPs and MPAs, the results of the questionnaire will be compared over two points in time.

- The quantitative and qualitative components (Between-method-Triangulation):

The qualitative and quantitative study parts are performed sequentially. The patient survey focus groups with patients are conducted before performing the questionnaire with patients (exploratory), and the qualitative interviews with GPs and MPAs are following the quantitative survey (explanatory).

Integration will take place when the results of the questionnaire lead to decisions about the interviews guide (GPs and MPAs), when the development of the patient questionnaire is based on the results of the focus groups with patients, and when the results are interpreted and compared between the study parts and between the groups. We adopt a model that relies on the principle of complementarity; using the strengths of one method to enhance the other [67].

We anticipate the possibility of conflicting findings between the surveys and interviews. This is to be expected to some extent simply because the methods are very different. By using the framework of Moffat et al., we will not use the results of the two methods for cross-validation purposes but will consider them to be complementary [67]. We will compare the demographic characteristics of the samples for differences. We will also critically appraise the use of quantitative measures. While qualitative interviews have the potential to explore some issues in greater depth than a quantitative questionnaire, they could also reveal some misunderstanding of the survey questions.

### Outcomes

The primary outcome is that the views of GPs, MPAs, and patients be obtained, on the supply and utilization of health services during the COVID-19 pandemic, addressing positive as well as negative aspects, such as:

- aspects of underutilization/undersupply;
- possible identification of services that turned out not to be necessary;
- unanticipated benefits like improvement of organizational matters and use of virtually-delivered care.

Piloting the structural development of the network, secondary outcomes are the number of participating GPs and MPAs and the number of participating patients. The number of participating GPs will be compared in terms of whether or not they are members of RESPoNsE.

### Data analysis

Data from the returned self-reported questionnaires will be entered manually into a Microsoft Excel spreadsheet and imported into IBM SPSS software (V.28) for data cleaning and analyses. Descriptive statistics (such as frequencies and proportions), summary statistics (such as means with standard deviations or median with interquartile range, as appropriate), and the number of missing values for each variable will be reported. Missing values will not be imputed.

The association of selected demographic and professional characteristics regarding respondents´ views and experiences will be verified with the chi-square test of independence for categorical variables and parametric or non-parametric tests for continuous variables, depending on the distribution. A comparison of GPs´and MPAs´ answers will be performed accordingly. The threshold for significance will be set at 0.05. Multivariate regression analyses will be applied to selected items where appropriate.

Qualitative data from the interviews and focus groups will be analyzed using thematic analyses based on a combined inductive and deductive approach [68]. Data analysis and coding will be facilitated by using the software MAXQDA 2022, release 22.1.1. One author of the multidisciplinary research team will start categorizing and coding the first interviews and develop a coding framework, which then will be discussed with the other members of the research team before being applied to the proceeding interviews.

### Dissemination of results

Results will be published in peer-reviewed open-access journals.

A symposium will be held, presenting and discussing the results with GPs, MPAs, members of the public, and representatives of political and professional decision-makers.

## Discussion

This project addresses the key role GPs and MPAs have in the care of patients during the pandemic, with or without COVID-19. They are usually the first point of contact and it is them who will be “left to manage the aftermath” [2], confirming their “first in, last out” role [3, 69].

The study will give GPs, MPAs, and patients the opportunity to voice their concerns, struggles, and needs during the COVID-19 pandemic. It will explore the impact of changing GP care during the pandemic, including lessons learned, and will help to assess which transformations might prevail [3, 70, 71].

The study will also provide a basis for further overdue discussions on reasonable appreciation and validation of the role of primary care during the pandemic, of the contribution of MPAs in a functioning GP care service, and support needs and how these can be addressed.

Integrating the patient perspective, the mixed quantitative and qualitative approach may also help to obtain a new view on previously perhaps overused care [72].

### Limitations

The COVID-19 pandemic so far has come in waves with very different demands on GP care. The current pandemic phase, with a dominating Omicron variant, and a seven-day-incidence of about 1,800 new cases per 100,000 inhabitants in Germany at the time of writing the protocol, may well have subsided by the time of data collection. The subjective impression of health care utilization and uptake may reflect the moment rather than the various phases of the pandemic.

The study is to be conducted in three federal states of Germany. Therefore, it might not represent the view of GPs, MPAs, and patients in other regions of Germany. On the other hand, the three regions compromise heterogenous economic and social conditions from metropolitan to rural areas.

A frequent problem in survey research on GPs is the low response rate. There is some controversy over the potential of response bias [73–75]. Several feasible strategies to improve the response rate failed to show an effect [56, 76]. We hope that, by keeping the questionnaires relatively short and addressing currently relevant topics, we attain a reasonable response rate.

## Study status

Ethics and data protection approvals have been obtained.

The questionnaire for GPs and MPAs has been constructed and piloted.

Data collection from the questionnaire for GPS and MPAs began in March 2022.

The patient focus groups were conducted in April 2022.

Interviews and the patient survey will be prepared and conducted over the next six months.

In 2023, the GP and MPA survey will be repeated.

## Ethical considerations

Ethical approval was obtained from the Ethic Commission of Charité–Universitaetsmedizin Berlin, Germany (EA2/303/21, January 20, 2022) and from Universitätsklinikum Jena, Thuringia (2022-2537-Bef, January 31, 2022).

## Data availability statement

The study will generate two kinds of data: the quantitative data of the surveys will be collected and analyzed anonymously. All variables will be reported descriptively with adequate summary measures. Original data will be available upon reasonable request. The qualitative data of the transcribed interviews´ data are more sensitive: before taking part, patients will give their written consent that these data may be used for the purpose of this study only. This data will not be shared beyond the research team.

## Supporting information

S1 File Response form. (PDF)

S2 File Original GP questionnaire (German). (PDF)

S3 File Original MPA questionnaire (German). (PDF)
