# Supplementary material for: Changes to utilization and provision of health care in German GP practices during the COVID 19-pandemic: Protocol for a mixed methods study on the viewpoint of GPs, medical practice assistants, and patients
Source: PLoS One. 2023 Apr 13;18(4):e0279413. doi: 10.1371/journal.pone.0279413 (PMC10101402; doi:10.1371/journal.pone.0279413)
Supplement: S1 File — (PDF) [file pone.0279413.s001.pdf]

Logo
RESPoNsE

Response Form about study participation

Fax: 030 – 450 7514 299
or Mail to forschungspraxennetz@charite.de

Name: ____

Practice Stamp

☐ **I completed the questionnaire and send it back by post**

☐ **I do not wish to take part in the survey**

☐ I am interested to become a member of the practice based research network and wish to be included into the mailing list for further information. My mail address is:

____

Further participation in this study:

☐ I am interested to take part in the interview study (one interview, duration 30-40 minutes)

☐ One of the MPAs of my practice is interested to take part in the interview study (one interview, duration 30-40 minutes)

☐ I am interested to take part in the patient survey and am happy to hand out questionnaires to the patients of my practice
