# Supplementary material for: Changes to utilization and provision of health care in German GP practices during the COVID 19-pandemic: Protocol for a mixed methods study on the viewpoint of GPs, medical practice assistants, and patients
Source: PLoS One. 2023 Apr 13;18(4):e0279413. doi: 10.1371/journal.pone.0279413 (PMC10101402; doi:10.1371/journal.pone.0279413)
Supplement: S2 File — (PDF) [file pone.0279413.s002.pdf]

Fragebogen für Ärzt*innen

Vielen Dank für Ihre Teilnahme an unserer Befragung!

Angaben zu Ihrer Person und Ihrer Praxis	
Geschlecht: ☐ Weiblich ☐ Männlich ☐ Divers	Alter: ____ Jahre
Bundesland:<br>☐ Berlin ☐ Brandenburg ☐ Thüringen ☐ Anderes	Praxisregion:<br>☐ eher ländlich ☐ eher städtisch
Tätigkeitsart: ☐ selbständig ☐ angestellt	Tätigkeitsumfang: ☐ Vollzeit ☐ Teilzeit
Praxisart:<br>☐ Einzelpraxis ☐ Gemeinschaftspraxis / BAG<br>☐ MVZ ☐ Praxisgemeinschaft	Anzahl Patient*innen pro Quartal pro Sitz:<br>IV. Quartal 21 ________<br>IV. Quartal 20 ________<br>IV. Quartal 19 ________

Patient*innenversorgung von NICHT an Covid-19 erkrankten Patient*innen während der Pandemie

Wie haben sich die Häufigkeiten durchgeführter Maßnahmen/Leistungen während der Corona-Pandemie verändert:

	Viel seltener	Etwas seltener	Weder noch	Etwas häufiger	Viel häufiger	Weiß ich nicht
Terminsprechstunden	☐	☐	☐	☐	☐	☐
Akutsprechstunden	☐	☐	☐	☐	☐	☐
Videosprechstunden	☐	☐	☐	☐	☐	☐
Telefonsprechstunden	☐	☐	☐	☐	☐	☐
Kurze telefonische Abklärung	☐	☐	☐	☐	☐	☐
Aufnahme neuer Patient*innen	☐	☐	☐	☐	☐	☐
Hausbesuche	☐	☐	☐	☐	☐	☐
Heimbesuche	☐	☐	☐	☐	☐	☐
DMP-Kontrollen	☐	☐	☐	☐	☐	☐
Patient*innen-Schulungen	☐	☐	☐	☐	☐	☐
Routine-Verlaufskontrollen (z.B. Blutdruck)	☐	☐	☐	☐	☐	☐
Gesundheitsuntersuchungen / Check-Up 35	☐	☐	☐	☐	☐	☐
Laboruntersuchungen (außer Covid-19)	☐	☐	☐	☐	☐	☐
Impfungen (außer Covid-19 und Influenza)	☐	☐	☐	☐	☐	☐
Sonstiges (Bitte ankreuzen und hier ergänzen):	☐	☐	☐	☐	☐	☐

Wenn Sie an Ihre **chronisch kranken Patient*innen** denken, die früher mehrmals im Quartal in die Praxis kamen – Welchen Aussagen stimmen Sie zu, wenn Sie die Corona-Pandemie mit der Zeit davor vergleichen?

	Stimme voll und ganz zu	Stimme zu	Weder noch	Stimme nicht zu	Stimme gar nicht zu	Weiß ich nicht
Wir haben diese Patient*innen in der Praxis gleich gut versorgt.	☐	☐	☐	☐	☐	☐
Ich habe gemerkt, dass sich der Gesundheitszustand mancher Patient*innen verschlechtert hat.	☐	☐	☐	☐	☐	☐
Ich habe gemerkt, dass sich der Gesundheitszustand mancher Patient*innen verbessert hat.	☐	☐	☐	☐	☐	☐
Ich habe versucht, Praxisbesuche dieser Patient*innen zu reduzieren	☐	☐	☐	☐	☐	☐
- durch gezielte Anrufe	☐	☐	☐	☐	☐	☐
- durch vermehrte Hausbesuche	☐	☐	☐	☐	☐	☐
- durch Videosprechstunden	☐	☐	☐	☐	☐	☐
Diese Patient*innen kamen von sich aus seltener in die Praxis.	☐	☐	☐	☐	☐	☐
Manche Maßnahmen/ Leistungen stellten sich während der Pandemie als verzichtbar heraus.	☐	☐	☐	☐	☐	☐

Ich finde es sinnvoll, folgende Leistungen auch nach der Pandemie seltener durchzuführen als vor der Pandemie:

Allgemeine Fragen zu Ihrer Arbeit während der Pandemie

Welchen der folgenden Aussagen stimmen Sie in Bezug auf Ihre Arbeit während der Pandemie zu?

	Stimme voll und ganz zu	Stimme zu	Weder noch	Stimme nicht zu	Stimme gar nicht zu
Meine Arbeit bereitet mir unter den jetzigen Umständen weiterhin Freude.	☐	☐	☐	☐	☐
Es wird von den Hausärzt*innen mehr erwartet als sie leisten können.	☐	☐	☐	☐	☐
Ich denke darüber nach, meinen Job zu wechseln.	☐	☐	☐	☐	☐
Es gibt mehr Konflikte im Team.	☐	☐	☐	☐	☐
Die Motivation im Team ist unverändert.	☐	☐	☐	☐	☐
Viele Patient*innen verhalten sich rücksichtsloser.	☐	☐	☐	☐	☐
Viele Patient*innen verhalten sich rücksichtsvoller.	☐	☐	☐	☐	☐
Ich muss für meine Arbeit einen erheblichen Mehraufwand leisten.	☐	☐	☐	☐	☐

Um die Versorgung unserer Patient*innen auch unter Pandemie-Bedingungen gut aufrechterhalten zu können, benötigen wir als Hausärzt*innen:

	Stimme voll und ganz zu	Stimme zu	Weder noch	Stimme nicht zu	Stimme gar nicht zu
Mehr Material (einschl. Schutzkleidung)	☐	☐	☐	☐	☐
Mehr Personal	☐	☐	☐	☐	☐
Besser qualifiziertes Personal	☐	☐	☐	☐	☐
Hilfreiche Informationen durch offizielle Organisationen zu Corona-Maßnahmen	☐	☐	☐	☐	☐
Einbeziehung in gesundheitspolitische Entscheidungen	☐	☐	☐	☐	☐
Eine Bezahlung des unvorhergesehenen Mehraufwandes	☐	☐	☐	☐	☐
Mehr Wertschätzung der geleisteten Arbeit	☐	☐	☐	☐	☐
- durch die Patient*innen	☐	☐	☐	☐	☐
- durch die Gesellschaft	☐	☐	☐	☐	☐
Sonstiges (Bitte ergänzen Sie!):					

Informationen während der Pandemie

Wie schätzen Sie zusammenfassend die Informationen während der Pandemie durch folgende Körperschaften / Verbände / Interessenvertretungen / Organisationen ein?

	Sehr hilfreich	Eher hilfreich	Weder noch	Eher nicht hilfreich	Gar nicht hilfreich	Weiß ich nicht
Kassenärztliche Vereinigung (KV)	☐	☐	☐	☐	☐	☐
Ärztekammer	☐	☐	☐	☐	☐	☐
Robert-Koch-Institut (RKI)	☐	☐	☐	☐	☐	☐
Ständige Impfkommission (STIKO)	☐	☐	☐	☐	☐	☐
Paul-Ehrlich-Institut (PEI)	☐	☐	☐	☐	☐	☐
Öffentliches Gesundheitswesen (z.B. Gesundheitsämter)	☐	☐	☐	☐	☐	☐
Hausärzteverband	☐	☐	☐	☐	☐	☐
Regierungsstellen	☐	☐	☐	☐	☐	☐
Bundeszentrale für gesundheitliche Aufklärung (BzgA)	☐	☐	☐	☐	☐	☐
Deutsche Gesellschaft für Allgemeinmedizin (DEGAM)	☐	☐	☐	☐	☐	☐
Eigenes professionelles Umfeld	☐	☐	☐	☐	☐	☐

Was hat Sie als Hausärzt*in während der Pandemie am meisten belastet? Bitte ergänzen Sie!
