# Supplementary material for: Changes to utilization and provision of health care in German GP practices during the COVID 19-pandemic: Protocol for a mixed methods study on the viewpoint of GPs, medical practice assistants, and patients
Source: PLoS One. 2023 Apr 13;18(4):e0279413. doi: 10.1371/journal.pone.0279413 (PMC10101402; doi:10.1371/journal.pone.0279413)
Supplement: S3 File — (PDF) [file pone.0279413.s003.pdf]

Fragebogen für MFA

Gerne bei mehreren MFA kopieren!

Vielen Dank für Ihre Teilnahme an unserer Befragung!

**Angaben zu Ihrer Person und Ihrer Arbeitsstelle**	
Geschlecht: ☐ Weiblich ☐ Männlich ☐ Divers	Alter: ____ Jahre
Bundesland:<br>☐ Berlin ☐ Brandenburg ☐ Thüringen ☐ Anderes	Praxisregion:<br>☐ eher ländlich ☐ eher städtisch
Praxisart:<br>☐ Einzelpraxis ☐ Gemeinschaftspraxis / BAG<br>☐ MVZ ☐ Praxisgemeinschaft	Tätigkeitsumfang:<br>☐ Vollzeit ☐ Teilzeit

Patient*innenversorgung von NICHT an Covid-19 erkrankten Patient*innen während der Pandemie

Wie haben sich die Häufigkeiten durchgeführter Maßnahmen/Leistungen während der Corona-Pandemie verändert:

	Viel seltener	Etwas seltener	Weder noch	Etwas häufiger	Viel häufiger	Weiß ich nicht
Terminsprechstunden	☐	☐	☐	☐	☐	☐
Akutsprechstunden	☐	☐	☐	☐	☐	☐
Videosprechstunden	☐	☐	☐	☐	☐	☐
Telefonsprechstunden	☐	☐	☐	☐	☐	☐
Kurze telefonische Abklärung	☐	☐	☐	☐	☐	☐
Aufnahme neuer Patient*innen	☐	☐	☐	☐	☐	☐
Hausbesuche	☐	☐	☐	☐	☐	☐
Heimbesuche	☐	☐	☐	☐	☐	☐
DMP-Kontrollen	☐	☐	☐	☐	☐	☐
Patient*innen-Schulungen	☐	☐	☐	☐	☐	☐
Routine-Verlaufskontrollen (z.B. Blutdruck)	☐	☐	☐	☐	☐	☐
Gesundheitsuntersuchungen / Check-Up 35	☐	☐	☐	☐	☐	☐
Laboruntersuchungen (außer Covid-19)	☐	☐	☐	☐	☐	☐
Impfungen (außer Covid-19 und Influenza)	☐	☐	☐	☐	☐	☐
Sonstiges (Bitte ankreuzen und hier ergänzen):	☐	☐	☐	☐	☐	☐

Wenn Sie an Ihre **chronisch kranken Patient*innen** denken, die früher mehrmals im Quartal in die Praxis kamen – Welchen Aussagen stimmen Sie zu, wenn Sie die Corona-Pandemie mit der Zeit davor vergleichen?

	Stimme voll und ganz zu	Stimme zu	Weder noch	Stimme nicht zu	Stimme gar nicht zu	Weiß ich nicht
Wir haben diese Patient*innen in der Praxis gleich gut versorgt.	☐	☐	☐	☐	☐	☐
Ich habe gemerkt, dass sich der Gesundheitszustand mancher Patient*innen verschlechtert hat.	☐	☐	☐	☐	☐	☐
Ich habe gemerkt, dass sich der Gesundheitszustand mancher Patient*innen verbessert hat.	☐	☐	☐	☐	☐	☐
Wir haben versucht, Praxisbesuche dieser Patient*innen zu reduzieren.	☐	☐	☐	☐	☐	☐
Diese Patient*innen kamen von sich aus seltener in die Praxis.	☐	☐	☐	☐	☐	☐
Manche Maßnahmen/ Leistungen stellten sich während der Pandemie als verzichtbar heraus.	☐	☐	☐	☐	☐	☐

Allgemeine Fragen zu Ihrer Arbeit während der Pandemie

Welchen der folgenden Aussagen stimmen Sie in Bezug auf Ihre Arbeit während der Pandemie zu?

	Stimme voll und ganz zu	Stimme zu	Weder noch	Stimme nicht zu	Stimme gar nicht zu
Meine Arbeit bereitet mir unter den jetzigen Umständen weiterhin Freude.	☐	☐	☐	☐	☐
Es wird von den MFA mehr erwartet als sie leisten können.	☐	☐	☐	☐	☐
Ich denke darüber nach, meinen Job zu wechseln.	☐	☐	☐	☐	☐
Es gibt mehr Konflikte im Team.	☐	☐	☐	☐	☐
Die Motivation im Team ist unverändert.	☐	☐	☐	☐	☐
Viele Patient*innen verhalten sich rücksichtsloser.	☐	☐	☐	☐	☐
Viele Patient*innen verhalten sich rücksichtsvoller.	☐	☐	☐	☐	☐
Ich muss für meine Arbeit einen erheblichen Mehraufwand leisten.	☐	☐	☐	☐	☐

Um die Versorgung unserer Patient*innen auch unter Pandemie-Bedingungen gut aufrechterhalten zu können, benötigen wir als MFA:

	Stimme voll und ganz zu	Stimme zu	Weder noch	Stimme nicht zu	Stimme gar nicht zu
Mehr Material (einschl. Schutzkleidung)	☐	☐	☐	☐	☐
Mehr Personal	☐	☐	☐	☐	☐
Besser qualifiziertes Personal	☐	☐	☐	☐	☐
Hilfreiche Informationen durch offizielle Organisationen zu Corona-Maßnahmen	☐	☐	☐	☐	☐
Einbeziehung in gesundheitspolitische Entscheidungen	☐	☐	☐	☐	☐
Eine Bezahlung des unvorhergesehenen Mehraufwandes	☐	☐	☐	☐	☐
Mehr Wertschätzung der geleisteten Arbeit	☐	☐	☐	☐	☐
- durch die Patient*innen	☐	☐	☐	☐	☐
- durch die Gesellschaft	☐	☐	☐	☐	☐
Sonstiges (Bitte ergänzen Sie!):					

Persönliche Sorgen und Ängste während der Pandemie

Welche der folgenden Dinge bereiten Ihnen seit der Pandemie Sorgen oder Ängste?

	Stimme voll und ganz zu	Stimme zu	Weder noch	Stimme nicht zu	Stimme gar nicht zu
Anhaltender Stress	☐	☐	☐	☐	☐
Eigene körperliche Gesundheit	☐	☐	☐	☐	☐
Eigene psychische Gesundheit	☐	☐	☐	☐	☐
Gefahr der eigenen Ansteckung	☐	☐	☐	☐	☐
Gefahr andere anzustecken	☐	☐	☐	☐	☐
Eigene Corona-Folgeerkrankungen (z.B. Long-Covid)	☐	☐	☐	☐	☐
Zu wenig Erholungsmöglichkeiten	☐	☐	☐	☐	☐
Zu wenig Zeit für die Familie	☐	☐	☐	☐	☐
Die allgemeine gesellschaftliche Entwicklung	☐	☐	☐	☐	☐
Drohungen durch Patient*innen	☐	☐	☐	☐	☐
Verbale Angriffe durch Patient*innen	☐	☐	☐	☐	☐
Körperliche Angriffe durch Patient*innen	☐	☐	☐	☐	☐
Wirtschaftliche Existenz der Praxis	☐	☐	☐	☐	☐

Was hat Sie als MFA während der Pandemie am meisten belastet? Bitte ergänzen Sie!
